# Metabolic Dysfunction‐Associated Steatotic Liver Disease in Adults With Acromegaly: A Meta‐Analysis of Observational Studies

**DOI:** 10.1002/dmrr.70231

**Published:** 2026-09-21

**Authors:** Alessandro Mantovani, Riccardo Morandin, Elisa Molinaroli, Dario Messetti, Nicoletta Rolli, Matteo Forti, Marta Zoco, Stergios A. Polyzos, Giovanni Targher

**Affiliations:** ^1^ Department of Medicine University of Verona Verona Italy; ^2^ Metabolic Diseases Research Unit IRCCS Sacro Cuore Don Calabria Hospital Negrar di Valpolicella Italy; ^3^ Laboratory of Pharmacology School of Medicine Aristotle University of Thessaloniki Thessaloniki Greece

**Keywords:** acromegaly, GH, growth hormone, IGF‐1, insulin‐like growth factor‐1, liver fat content, liver fibrosis, MASLD

## Abstract

**Aims:**

Acromegaly is a chronic, progressive disease characterised by excess growth hormone secretion and elevated circulating insulin‐like growth factor 1 levels. Data on hepatic fat content and liver stiffness in adults with active or controlled acromegaly remain inconsistent.

**Methods:**

We systematically searched PubMed and Scopus from inception through 15 June 2026 to identify studies comparing hepatic steatosis and liver stiffness in adults with acromegaly and in controls. Hepatic fat content was quantified using magnetic resonance imaging (MRI)‐based techniques, and liver stiffness was assessed using vibration‐controlled transient elastography or other elastography methods. Pooled effect sizes were reported as weighted mean differences (WMDs) with 95% confidence intervals.

**Results:**

We identified 9 observational studies, including 190 patients with acromegaly and 235 age‐, sex‐, and BMI‐matched controls. Compared with controls, patients with acromegaly had significantly lower hepatic fat content, as assessed by MRI‐based techniques (six studies; random‐effects WMD −3.04% [95% CI −4.81 to −1.26%]; *I* ^2^ = 43.7%). This difference remained significant in patients with active acromegaly (6 studies; WMD −2.96% [95% CI −4.30 to −1.62]; *I* ^2^ = 0%), but not in those with controlled acromegaly (four studies; WMD −2.57% [95% CI −6.72 to 1.59]; *I* ^2^ = 70.3%). Liver stiffness measurements did not differ between patients with acromegaly and control subjects (four studies; WMD 0.94 kPa [95% CI −0.45 to 2.33], *I* ^2^ = 87.9%). Sensitivity analyses yielded consistent findings.

**Conclusions:**

Our meta‐analysis suggests that patients with acromegaly have significantly lower hepatic fat content than controls, particularly among those with active disease, whereas liver stiffness does not appear to differ significantly between the groups.

## 1 Introduction

Metabolic dysfunction‐associated steatotic liver disease (MASLD) is the leading cause of chronic liver disease worldwide [1]. It represents a major public health challenge due to its rapidly rising global prevalence and the substantial burden of hepatic and cardiovascular morbidity and mortality [2, 3, 4, 5]. MASLD is estimated to affect approximately 35%–40% of the general adult population [6], nearly 65%–70% of individuals with type 2 diabetes mellitus (T2DM) [7], and up to 80%–85% of those with obesity [8].

The pathophysiology of MASLD is complex and reflects the interplay among genetic susceptibility, metabolic dysfunction, and environmental and lifestyle factors [9, 10]. The “multiple‐hit” hypothesis postulates that multiple pathogenic mechanisms act simultaneously to drive the development and progression of MASLD. These include insulin resistance, adipose tissue dysfunction, lipotoxicity, low‐grade chronic inflammation, altered gut microbiota, and endocrine disturbances [9, 10]. In addition to obesity and T2DM [11], other endocrine disorders have been associated with a higher likelihood of MASLD, including primary hypothyroidism, polycystic ovary syndrome, and growth hormone deficiency (GHD) [12, 13, 14, 15]. In a recent meta‐analysis of 18 observational studies, our group also found that circulating GH and insulin‐like growth factor‐1 (IGF‐1) levels are inversely associated with the presence and severity of MASLD [16].

Acromegaly is the endocrine counterpart of adult GHD [17]. Acromegaly is a rare, chronic, progressive disease characterised by excess GH secretion and elevated circulating IGF‐1 levels, most commonly caused by a GH‐secreting pituitary adenoma [17]. Clinically, it presents with progressive somatic overgrowth, including enlargement of the hands and feet, coarse facial features, mandibular prognathism, macroglossia, and widening of the interdental spaces [17]. Patients with acromegaly often develop insulin resistance, prediabetes or T2DM, hypertension, and atherogenic dyslipidemia, all well‐established risk factors for MASLD [17].

Despite this adverse metabolic risk profile, the relationship between acromegaly and MASLD remains controversial. GH exerts potent lipolytic effects, reducing adipose tissue mass, while promoting hepatic insulin resistance and increasing hepatic glucose production [13, 18]. Conversely, IGF‐1 has insulin‐sensitising, anti‐inflammatory, and potentially anti‐fibrotic effects [13, 18]. Moreover, patients with acromegaly typically exhibit reduced visceral adiposity despite insulin resistance [17], suggesting a unique metabolic phenotype that may differentially influence hepatic fat accumulation and liver fibrosis. The complex and sometimes opposing actions of GH and IGF‐1 on lipid metabolism, insulin sensitivity, and hepatic fibrogenesis make the net effect of acromegaly on liver health difficult to predict. Observational studies have reported low rates of hepatic steatosis in patients with active acromegaly, whereas liver fibrosis has been studied less extensively and with inconsistent findings [19]. Additionally, biochemical control of acromegaly reduces the lipolytic and hepatic effects of excess GH, often leading to increased body weight and greater hepatic fat accumulation [19].

Given the conflicting evidence on hepatic steatosis (MASLD) in patients with acromegaly, we conducted a systematic review and meta‐analysis of observational studies to quantitatively assess differences in hepatic fat content and liver stiffness between adults with acromegaly (active vs. controlled disease) and control subjects.

## 2 Materials and Methods

### 2.1 Protocol Registration

The protocol for this systematic review was registered in advance on the Open Science Framework (OSF) registry (Registration DOI: 10.17605/OSF.IO/BYRHV).

### 2.2 Data Sources and Search Strategy

This study was conducted in accordance with the Meta‐analysis Of Observational Studies in Epidemiology (MOOSE) guidelines [20, 21]. We systematically searched PubMed and Scopus from database inception through 15 June 2026 to identify observational studies comparing hepatic fat content, quantified by magnetic resonance spectroscopy (MRS) or magnetic resonance imaging‐proton density fat fraction (MRI‐PDFF), and liver stiffness measurements, assessed by magnetic resonance elastography (MRE), shear wave elastography (SWE), or vibration‐controlled transient elastography (VCTE), in patients with active or controlled acromegaly versus control subjects.

The search strategy, using both medical subject headings (MeSH) and free‐text terms, was as follows: (‘acromegaly’ AND ‘fatty liver’), (‘acromegaly’ AND ‘nonalcoholic fatty liver disease’), (‘acromegaly’ AND ‘nonalcoholic steatohepatitis’), (‘Acromegaly’ AND ‘NAFLD’), (‘acromegaly’ AND ‘NASH’), (‘acromegaly’ AND ‘metabolic dysfunction associated steatotic liver disease’), (‘acromegaly’ AND ‘metabolic associated steatohepatitis’), (‘acromegaly’ AND ‘MASLD’), (‘acromegaly’ AND ‘MASH’).

Additionally, we used the restriction terms ‘human’ and ‘English’. The syntax for database searches on PubMed and Scopus is provided in Supporting Information S1: Table S1. An additional search was conducted by reviewing the reference lists of papers included in previous reviews [19].

### 2.3 Inclusion and Exclusion Criteria

We included observational studies comparing hepatic fat content, assessed by MRS or MRI‐PDFF, and liver stiffness, assessed by VCTE, SWE, or MRE, in adults (> 18 years) with active or controlled acromegaly versus controls. We excluded studies that met any of the following criteria: (a) absence of a control group; (b) assessment of hepatic steatosis using liver ultrasonography, controlled attenuation parameter (CAP measured using FibroScan), serum liver enzyme levels, or other surrogate biomarkers (e.g., fatty liver index); (c) excessive alcohol consumption (> 20 g/day in women and more than 30 g/day in men) and other known causes of liver disease; (d) paediatric populations (age  < 18 years); and (e) reviews, case reports, editorials, or commentaries.

### 2.4 Data Extraction and Quality Assessment

Two investigators (R.M and A.M) independently screened the titles and abstracts of all identified records against the predefined inclusion criteria. Full‐text reviews of potentially eligible studies were then independently conducted by both investigators. Any disagreements about study inclusion were resolved by consulting a third investigator (G.T).

For each eligible study, the following data were extracted: author, publication year, country, sample size, demographic characteristics (age, sex, body mass index [BMI]), homoeostasis model assessment–insulin resistance (HOMA–IR score), hepatic fat content, liver stiffness measurement, and circulating IGF‐1 and/or GH levels.

The methodological quality of the included studies was assessed using the Newcastle–Ottawa Scale (NOS), with versions adapted for case‐control studies [22, 23]. The NOS evaluates study quality across three domains (selection, comparability, and outcome/exposure) and provides a maximum score of 9 stars for case‐control studies [22, 23]. Studies were classified as high quality if they achieved 8‐9 stars, moderate quality if they scored 5‐7 stars, and low quality if they scored 0‐4 stars. Quality assessment was independently performed by two investigators (R.M. and A.M.). Any discrepancies were resolved through discussion and, when necessary, consultation with a third investigator (G.T.).

### 2.5 Data Synthesis and Statistical Analysis

The primary outcome of the study was the difference in hepatic fat content (measured by MRI‐based techniques) between adult patients with active or controlled acromegaly and control subjects. The secondary outcome was the difference in liver stiffness measurement between patients with active or controlled acromegaly and controls. In two studies, when needed, the data of interest were estimated by directly extracting values from the published boxplots using WebPlotDigitizer (https://automeris.io). When data were reported as medians ± interquartile ranges, hepatic fat content (or liver stiffness) was converted to means ± SD using mathematical formulas [24].

Effect sizes were expressed as weighted mean differences (WMDs) with corresponding 95% confidence intervals (95% CIs). The robustness of the pooled estimates was evaluated using leave‐one‐out sensitivity analyses, in which each study was omitted sequentially, and the meta‐analysis was rerun. We performed univariable meta‐regression analyses to assess whether age, sex, BMI, and HOMA‐IR score influenced the WMDs for MRI‐assessed hepatic fat content comparing patients with active or controlled acromegaly to control subjects. We also conducted a meta‐regression assessing the association between circulating IGF‐1 levels and WMDs in hepatic fat content, comparing patients with active acromegaly to those with controlled acromegaly. Standardised mean difference (SMD) analyses were also performed as sensitivity analyses to assess the robustness of the findings across MRI‐ and LSM‐based techniques, which may have different measurement variability.

Statistical heterogeneity was initially assessed by visual inspection of forest plots and then quantified using Cochran's *Q* (chi‐square) test and the *I* ^2^‐statistic. Heterogeneity was considered low when *I* ^2^‐values ranged from 0% to 50% and substantial when *I* ^2^‐values exceeded 50%. Publication bias was assessed using Doi plot, a graphical tool that detects publication bias by plotting the effect size (ES) (on the *X*‐axis) against the absolute *Z*‐score (on the *Y*‐axis) in reverse order. The Doi plot provides an alternative to funnel plots, especially when the number of eligible studies is fewer than 10 [25].

All statistical analyses were two‐sided, and *p*‐values < 0.05 were considered statistically significant. Analyses were conducted in R software (version 4.4.1, R Foundation for Statistical Computing, Vienna, Austria) using the *meta* (version 8.0–1), *metafor* (version 4.6–0), and *metasens* (version 1.5–3) packages.

## 3 Results

Supporting Information S1: Figure S1 shows a PRISMA flow diagram summarising the search and selection processes for the meta‐analysis. After reviewing titles and abstracts and removing duplicates, we identified 11 potentially eligible studies in PubMed and Scopus, spanning inception to 15 June 2026. We subsequently excluded two studies [26, 27], as specified in Supporting Information S1: Table S2. Consequently, we identified 9 observational studies [28, 29, 30, 31, 32, 33, 34, 35, 36]: 6 studies comparing MRI‐assessed hepatic fat content between patients with active or controlled acromegaly and control subjects [25, 26, 27, 28, 29, 30] and three studies comparing liver stiffness measurements [34, 35, 36]. The study by Eroglu et al. contributed to both analyses [32].

Among studies assessing differences in MRI‐detected hepatic fat content between patients with active or controlled acromegaly and control subjects, a total of 176 individuals (53.4% men, age 47 ± 2 years, BMI 28.6 ± 1.76 kg/m^2^, HOMA‐IR 1.67 ± 0.42) were included in the final analysis. Of these, 89 were patients with acromegaly (61 of whom were also classified as having controlled acromegaly after pituitary surgery and/or medical therapy), and 87 were age‐ and sex‐matched controls. Table 1 summarises the main characteristics of the six studies included in the analysis. These studies were conducted in the United States (*n* = 2), Austria (*n* = 2), Denmark (*n* = 1), and Turkey (*n* = 1). All studies had a case‐control design, and control subjects were at least matched for age and sex to patients with acromegaly. Of the six studies, four also assessed hepatic fat content in patients with controlled acromegaly and compared it with that in control subjects. Disease control in acromegaly was achieved through pituitary surgery and/or medical therapies (i.e., GH receptor antagonists or somatostatin analogues) in all studies, except for the study by Winhofer et al. [28], in which disease control was obtained exclusively by surgery. According to the NOS, 4 studies received 7 stars and 2 received 6 stars (Supporting Information S1: Table S3).

**TABLE 1:** Observational studies comparing hepatic fat content, quantified non‐invasively by magnetic resonance spectroscopy (MRS) or MRI‐proton density fat fraction (MRI‐PDFF), among patients with active or controlled acromegaly and control subjects.

Author (year), country (ref.)	Study characteristics (*n*)	Acromegaly status	Hepatic fat content in acromegaly group (mean ± SD)	Hepatic fat content in control group (mean ± SD)	Mean age (years)	Mean BMI (kg/m^2^)	Male percentages (%)	Mean HOMA‐IR score	Serum IGF‐1 levels in acromegaly group (ng/mL) (mean ± SD)	Serum IGF‐1 levels in control group (ng/mL) (mean ± SD)	Serum GH levels in acromegaly group (ng/mL) (mean ± SD)	Serum GH levels in control group (ng/mL) (mean ± SD)
Winhofer et al. (2014)^a^, Austria [28]	Case‐control: 10 patients with active acromegaly versus 12 healthy controls matched for age, BMI, and sex	Active	MRS: 1.2 ± 1.2	MRS: 4.3 ± 4.5	45	27.3	54	ND	890 ± 437	ND	ND	ND
	7 patients with controlled acromegaly versus 12 healthy controls	Controlled (surgery)	MRS: 1.0 ± 0.6	MRS: 4.3 ± 4.5	45	27.3	54	ND	285 ± 93	ND	ND	ND
Bredella et al. (2017), USA [29]	Case‐control: 20 patients with active acromegaly versus 20 healthy controls matched for age, BMI, and sex	Active	MRS: 2.3 ± 2.2	MRS: 10 ± 14.8	47	30.5	50	Acromegaly group: 2.32 ± 1.3; Control group: 1.39 ± 2.29	842 ± 237	164.3 ± 51	7.8 ± 3.7	0.06 ± 0.04
	16 patients with controlled acromegaly versus 20 healthy controls	Controlled (surgery or medical therapies)	MRS: 3.0 ± 1.5	MRS: 10 ± 14.8	47	29.5	56	Acromegaly group: 0.97 ± 0.63; Control group: 1.39 ± 2.29	252 ± 67	ND	0.23 ± 0.15	0.06 ± 0.04
(Fellinger et al. 2020), Austria [30]	Case‐control: 15 patients with active acromegaly versus 17 healthy controls matched for age, BMI and body composition	Active	MRS: 1.5 ± 1.6	MRS: 5.6 ± 8.1	46	26.7	56	Acromegaly group: 1.72 ± 0.95; Control group: 1.02 ± 0.38	ND	ND	ND	ND
Kukerbet al. (2021)^b^, USA [31]	Case‐control: 8 patients with active acromegaly versus 7 healthy controls matched for age and sex	Active	MRS: 2.5 ± 3.2	MRS: 5.9 ± 8.6	48	29.9	62	Acromegaly group: 3.15 ± 3.1	ND	ND	ND	ND
Eroglu et al. (2023), Turkey [32]	Case‐control: 15 patients with active pre‐ pregvisomant acromegaly versus 19 healthy controls matched for age, BMI, and sex	Active	MRI‐PDFF: 1.9 ± 0.9	MRI‐PDFF: 4.0 ± 3.4	47	29.5	56	Acromegaly group: 2.02 ± 1.04; Control group: 1.33 ± 0.65	546 ± 217	157 ± 76	ND	ND
	17 patients with controlled acromegaly versus 19 healthy controls	Controlled (surgery)	MRI‐PDFF: 5.9 ± 5.3	MRI‐PDFF: 4.0 ± 3.4	47	29.5	56	Acromegaly group: 2.07 ± 1.47; Control group: 1.33 ± 0.65	221 ± 91	ND	ND	ND
Arlien‐søborg et al. (2023), Denmark [33]	Case‐control: 21 patients with active acromegaly versus 12 healthy controls matched for age and sex	Active	MRS: 2.1 ± 2.4	MRS: 11.2 ± 18	50	27	51	Acromegaly group: 3.13 ± 1.25; Control group: 1.80 ± 1.92	195 ± 51	ND	5.90 ± 2.9	0.52 ± 0.33
	21 patients with controlled acromegaly versus 12 healthy controls	Controlled (surgery or medical therapies)	MRS: 5.7 ± 6.2	MRS: 11.2 ± 18	50	27	51	Acromegaly group: 1.14 ± 0.53; Control group: 1.80 ± 1.92	ND	ND	ND	ND

Abbreviations: BMI, body mass index; GH, growth hormone; HOMA‐IR, homoeostasis model assessment of insulin resistance; IGF‐1, insulin‐like growth factor‐1; ND, not determined.

^a^ The study by Winhofer et al. [28] used a euglycemic clamp to assess insulin resistance.

^b^ Data from acromegalic patients treated with pegvisomant could not be included because variance measures were not reported. Studies in the table are ordered by publication year.

Among studies assessing liver stiffness in patients with acromegaly and control subjects, a total of 285 individuals (118 patients with acromegaly and 167 age‐, sex‐, and BMI‐matched controls; age 42 ± 4 years, BMI 29.5 ± 1.8 kg/m^2^, 50% men) were included in the final analysis. Table 2 summarises the main characteristics of the studies included in the analysis. All four studies were conducted in Turkey. One study included patients with controlled acromegaly, another included patients with active and controlled acromegaly, and the remaining two enroled patients with active acromegaly. According to the NOS, one study received 7 stars, and three studies received 6 stars (Supporting Information S1: Table S3).

**TABLE 2:** Observational studies comparing liver stiffness measurement assessed by magnetic resonance elastography (MRE), shear wave elastography (SWE), or vibration‐controlled transient elastography (VCTE) in patients with acromegaly and control subjects.

Author (year) country (ref.)	Study characteristics (n)	Methods for assessing LSM	LSM in acromegaly group (mean ± SD)	LSM in control group (mean ± SD)	Mean age (years)	Mean BMI (kg/m^2^)	Male percentage (%)	Serum IGF‐1 levels in acromegaly group (ng/mL) (mean ± SD	Serum IGF‐1 levels in control group (ng/mL) (mean ± SD)
Bankir et al. (2019), Turkey [34]	Case control study: 20 patients with controlled acromegaly, 20 patients with active acromegaly versus 20 healthy control subjects matched for age, sex and body surface area	pSWE	5.01 ± 1.24 for patients with controlled acromegaly; 6.50 ± 3.1 for those with active acromegaly	3.18 ± 1.22	42	28.5	65	178 ± 38 for patients with controlled acromegaly; 442 ± 152 for those with active acromegaly	73.4 ± 7.9
Eroglu et al. (2023), Turkey [32]	Case control study: 17 patients with controlled acromegaly versus 19 healthy controls matched for age, BMI, and sex	MRE	2.3 ± 0.2	2.2 ± 0.4	47	30.7	50	545 ± 217	157 ± 76
Coskum et al. (2024), Turkey [35]	Case control study: 23 patients with acromegaly versus 46 controls matched for age, sex, BMI and waist circumference	SWE	6.1 ± 1.5	5.1 ± 0.8	41	27.5	39	ND	ND
Apaydin et al. (2026), Turkey [36]	Case control study: 58 patients with acromegaly versus 82 healthy controls matched for age and sex	VCTE	4.7 ± 1.4	4.8 ± 1.8	38	31.3	48	ND	ND

*Note:* Studies in the table are ordered by publication year.

Abbreviations: BMI, body mass index; GH, growth hormone; HOMA‐IR, homoeostasis model assessment of insulin resistance; IGF‐1, insulin‐like growth factor‐1; LSM, liver stiffness measurement; ND, not determined; pSWE, point shear wave elastography.

**FIGURE 1:**
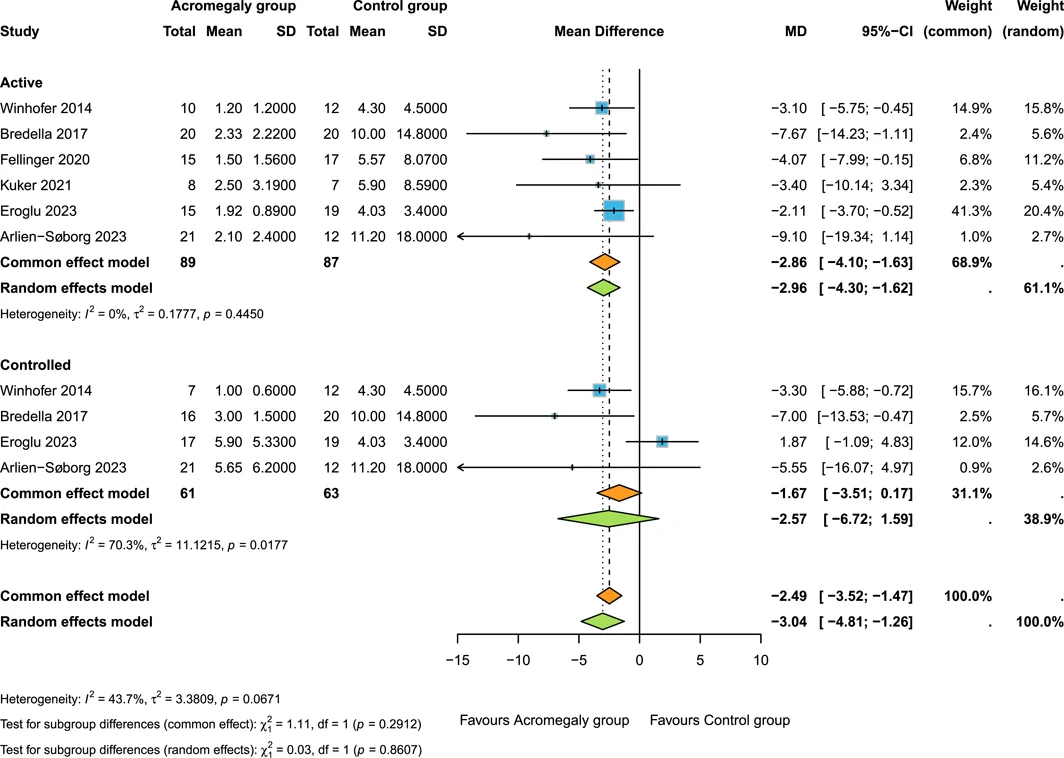
Forest plot and pooled estimates of weighted mean differences (WMDs) in hepatic fat content (assessed by magnetic resonance spectroscopy or magnetic resonance imaging‐proton density fat fraction) among patients with acromegaly and control subjects, stratified by acromegaly status (active vs. controlled disease).

Figure 1 shows the WMDs in MRI‐measured hepatic fat content between patients with acromegaly and matched control subjects, stratified by acromegaly status (active vs. controlled). Patients with acromegaly had significantly lower hepatic fat content than control subjects (random‐effects WMD −3.04% [95% CI −4.81; −1.26], *I* ^2^ = 43.7%). After stratification by acromegaly status, the differences remained significant only in patients with active acromegaly (random‐effects WMD −2.96% [95% CI −4.30; −1.62], *I* ^2^ = 0%), but not in those with controlled acromegaly (WMD −2.57% [95% CI −6.72; 1.59], *I* ^2^ = 70.3%), in whom hepatic fat content increased after pituitary surgery and/or medical therapies. The test for subgroup differences (random effects) was not statistically significant (*p* = 0.861). Results were unchanged when we repeated the analyses using SMDs instead of WMDs. Overall, patients with acromegaly had significantly lower hepatic fat content than control subjects (random‐effects SMD −0.56 [95% CI −0.83; −0.29]; *I* ^2^ = 13.6%). After stratification by disease activity, the difference remained significant in patients with active acromegaly (SMD −0.74 [95% CI −1.05; −0.43]; *I* ^2^ = 0%), but not in patients with controlled acromegaly (SMD −0.34 [95% CI −0.89; 0.22]; *I* ^2^ = 55.7%).

**FIGURE 2:**
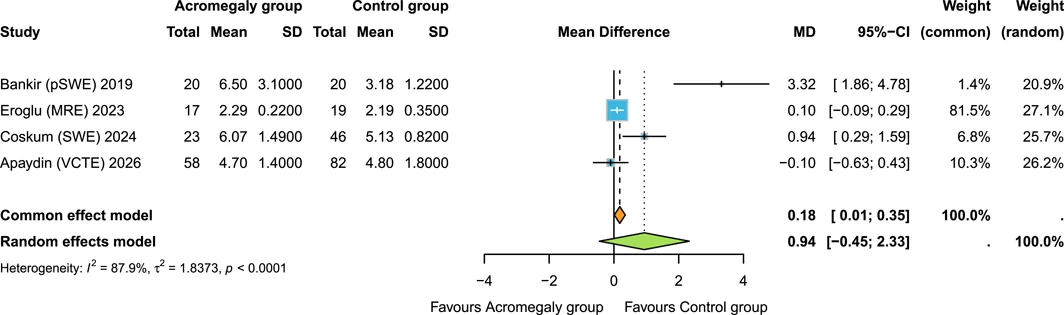
Forest plot and pooled estimates of weighted mean differences (WMDs) in liver stiffness measurement (assessed by vibration‐controlled transient elastography, shear wave elastography, or magnetic resonance elastography) among patients with acromegaly and control subjects.

Figure 2 shows the WMDs for liver stiffness measurements comparing patients with acromegaly and matched control subjects. Liver stiffness measurements did not significantly differ between patients with acromegaly and controls (random‐effects WMD 0.94 kPa [95% CI −0.45 to 2.33], *I* ^2^ = 87.9%). Repeating the analyses using SMDs instead of WMDs did not substantially change the results (SMD 0.59 [95% CI −0.03% –to 1.21%]; *I* ^2^ = 76.4%). In the study by Bankir et al. [34], replacing liver stiffness measurements from patients with active acromegaly with those from patients with controlled acromegaly yielded comparable results (data not shown).

### 3.1 Sensitivity Analyses and Univariable Meta‐Regressions

A leave‐one‐out sensitivity analysis assessing the influence of each study on the overall effect size showed that removing any study from the pooled primary analysis did not significantly affect WMDs for hepatic fat content and liver stiffness between patients with acromegaly and control subjects (Supporting Information S1: Figure S2). In univariable meta‐regression analyses, age (*β* = −0.390, 95% CI: −1.89–1.12, *p* = 0.612) (Supporting Information S1: Figure S3), BMI (*β* = 0.602, 95% CI: −0.45–1.66, *p* = 0.264) (Supporting Information S1: Figure S4), male sex (*β* = −0.217, 95% CI: −61.34–60.90, *p* = 0.994) Supporting Information S1: Figure S5), and HOMA‐IR (*β* = −1.204, 95% CI: −11.69–9.29, *p* = 0.822) (Supporting Information S1: Figure S6) did not significantly influence WMDs in hepatic fat content between patients with acromegaly and controls. Notably, restricting univariable meta‐regression analyses to studies (*n* = 3) with available data on circulating IGF‐1 levels in patients with active and controlled acromegaly (following pituitary surgery and/or medical therapies), we found that circulating IGF‐1 levels significantly influenced WMDs in hepatic fat content between patients with active and those with controlled acromegaly (*β* = 0.021, 95% CI: 0.007–0.033, *p* = 0.002) (Supporting Information S1: Figure S7).

### 3.2 Publication Bias

In studies comparing MRI‐detected hepatic fat content among patients with active or controlled acromegaly and control subjects, the Doi plot showed asymmetry, with a Luis Furuya–Kanamori (LFK) index of −3.44, indicating small‐study effects or possible publication bias (Supporting Information S1: Figure S8).

## 4 Discussion

The main and novel findings of this meta‐analysis, which included 9 observational studies involving adults with acromegaly and control subjects, are as follows: (a) compared with controls, patients with acromegaly had significantly lower hepatic fat content as assessed by MRI‐based techniques (random‐effects WMD −3.04% [95% CI −4.81; −1.26], *I* ^2^ = 43.7%); (b) after stratification by disease activity, the observed differences in hepatic fat content remained significant in patients with active acromegaly (WMD −2.96% [95% CI −4.30; −1.62], *I* ^2^ = 0%), but not in those with controlled disease, in whom hepatic fat content increased after pituitary surgery and/or medical therapies (WMD −2.57% [95% CI −6.72; 1.59], *I* ^2^ = 70.3%); (c) liver stiffness measurements, as assessed by VCTE SWE or MRE, did not significantly differ between patients with acromegaly and control subjects; (d) univariable meta‐regression analyses revealed that age, male sex, BMI, and HOMA‐IR did not significantly influence between‐group differences in hepatic fat content; and (e) in meta‐regression analyses restricted to studies with available data on circulating IGF‐1 levels in patients with active and controlled acromegaly, circulating IGF‐1 levels significantly influenced WMDs in hepatic fat content between patients with active and controlled acromegaly.

To our knowledge, this is the first meta‐analysis to quantitatively assess differences in hepatic fat content (measured by MRS and MRI‐PDFF) among patients with active acromegaly, controlled acromegaly, and control subjects. A recent scoping review summarised the available evidence on hepatic steatosis and fibrosis (using ultrasonography, VCTE, and MRI‐based techniques) in patients with active and controlled acromegaly [19]. However, due to its methodological design, it provided only a qualitative synthesis without pooling effect estimates and did not formally assess the magnitude of between‐group differences or explore potential sources of heterogeneity [19]. In contrast, we applied more stringent inclusion criteria, focussing exclusively on studies that quantified hepatic fat content using MRI‐based techniques, currently regarded as the non‐invasive reference standard for liver fat assessment [37], and on studies assessing liver stiffness using VCTE or other elastography methods, all of which are well‐validated non‐invasive techniques for liver fibrosis assessment [37]. By quantitatively pooling these more homogeneous data, we provided more robust estimates of the association among acromegaly, disease activity, and hepatic fat content, while also assessing liver stiffness measurements and exploring potential sources of heterogeneity using meta‐regression analyses.

Our findings indicate that adults with active acromegaly had significantly lower hepatic fat content than control subjects, whereas no significant difference in hepatic fat content was observed between patients with successfully treated acromegaly and control subjects. These findings align with those of the aforementioned scoping review [19], which identified a similar pattern of lower hepatic fat content during active disease and a potential increase after disease control. However, that review highlighted substantial heterogeneity across the available studies and emphasised the lack of quantitative evidence [19]. Notably, our meta‐analysis addressed this gap by quantifying the reduction in hepatic fat content among patients with active and controlled acromegaly and by showing that liver stiffness measurements do not differ significantly from those of control subjects. The patterns of hepatic fat content we observed in this meta‐analysis among patients with active and controlled acromegaly further corroborate the conclusions of our recently published review, which showed that MASLD is much more prevalent in adults with GHD than in control subjects and that recombinant GH (rhGH) treatment may have favourable effects on hepatic steatosis in adults with GHD [13].

From a pathophysiological perspective, GH exerts direct effects on hepatocytes by activating the Janus Kinase 2 (JAK2)—Signal Transducer and Activator of Transcription 5 (STAT5) signalling pathway, while its indirect effects on hepatic stellate cells are mediated predominantly by IGF‐1 [13, 18, 38, 39]. Under physiological conditions, GH exerts anti‐steatotic and possibly anti‐inflammatory and anti‐fibrotic actions in the liver, promotes hepatic glucose output by stimulating glycogenolysis and gluconeogenesis, reduces hepatic free fatty acid uptake and de novo lipogenesis, increases mitochondrial fatty acid oxidation, and facilitates very low‐density lipoprotein (VLDL) secretion [13, 18, 38, 39, 40]. Reduced GH/IGF‐1 axis activity after surgical or medical treatment of acromegaly may promote hepatic fat accumulation by attenuating the lipolytic and lipid‐oxidative effects of GH on adipose tissue and the liver [13, 18, 38, 39, 40]. In addition, biochemical control of acromegaly is associated with reduced lipolytic and hepatic effects of excess GH, often resulting in increased body weight and greater hepatic fat content [13, 18, 38, 39, 40]. In this context, normalisation of excess GH may paradoxically shift systemic metabolism towards greater hepatic lipid storage. Although these findings should be interpreted with caution, our meta‐regression analyses suggest that circulating IGF‐1 levels are associated with differences in hepatic fat content between patients with active and controlled acromegaly. However, this association should not be taken as evidence of a direct causal role for IGF‐1 in hepatic fat accumulation. Hepatocytes are the primary source of circulating IGF‐1 and do not express functional IGF‐1 receptors [13]. Therefore, circulating IGF‐1 may act primarily as a marker of GH activity rather than as a direct mediator of hepatic steatosis. In this context, higher GH levels may drive both increased hepatic fat accumulation and increased hepatic IGF‐1 production, thereby explaining the observed association between circulating IGF‐1 concentrations and hepatic fat content across different disease states.

As reported in Table 1, active acromegaly is characterised by insulin resistance (assessed by HOMA‐IR in all studies, except Winhofer et al. [28], who used a euglycemic clamp), despite low hepatic fat content. This suggests a dissociation among insulin resistance, visceral adiposity, and hepatic fat accumulation, and indicates that GH excess may profoundly influence insulin sensitivity and hepatic lipid metabolism in opposing directions [13, 18, 19]. The mechanisms underlying this apparent paradox remain poorly understood although some hypotheses have been proposed [19, 40, 41, 42, 43]. Experimentally, GH excess enhances hepatic mitochondrial activity, thereby increasing fatty acid *β*‐oxidation and overall energy expenditure, potentially limiting hepatic fat accumulation despite systemic insulin resistance [13, 18, 19]. In line with these metabolic adaptations, active acromegaly is also characterised by increased lean body mass and total body water, together with reductions in visceral and subcutaneous adipose tissue [19, 44]. Another proposed mechanism is a preferential redistribution of lipids towards skeletal muscle rather than the liver, with increased intramyocellular lipid deposition potentially contributing to the insulin resistance observed in active disease [19, 40, 41, 42].

The findings of our meta‐analysis may have important clinical implications. Clinicians should be aware that successful biochemical control of acromegaly can be accompanied by a relative increase in hepatic fat accumulation, likely reflecting the normalisation of GH‐dependent lipid metabolism rather than a deterioration in metabolic health per se. To date, only an open‐label randomised controlled trial has assessed the effect of acromegaly treatment on hepatic fat content measured by MRI. In this trial, 18 patients with well‐controlled acromegaly receiving somatostatin analogue monotherapy were randomised to continue their somatostatin analogue treatment or to receive combination therapy with pegvisomant *plus* a reduced dose of somatostatin analogue for 6 months [45]. Compared with continued somatostatin analogue monotherapy, combination therapy significantly increased hepatic fat content and reduced intramyocellular lipid content, suggesting that blocking GH may differentially affect lipid partitioning across metabolic tissues [45]. Nevertheless, the clinical implications of these findings remain unclear, given the lack of long‐term data linking treatment‐induced changes in hepatic fat content to hepatic inflammation, fibrosis progression, or liver‐related clinical events. Other small case series suggest that surgical removal of GH‐secreting pituitary adenomas is associated with higher hepatic fat content after 1.0–2.5 years of follow‐up [42, 46, 47]. Finally, the absence of significant differences in liver stiffness measurements between patients with acromegaly and control subjects may suggest that increased hepatic fibrosis is not a consistent feature of the disease, partly because hepatic steatosis is less common in patients with acromegaly; however, this conclusion should be further confirmed in larger follow‐up studies.

Our meta‐analysis has limitations inherent in the eligible studies. First, the observational, case‐control design of the included studies precludes causal inference and the establishment of temporal relationships. Our meta‐analysis relied on unadjusted summary data comparing hepatic fat content or liver stiffness between patients with acromegaly and control subjects (although most studies included control groups that were at least matched for age and sex and, in most cases, for BMI). In this sense, the pooled estimates may be susceptible to residual confounding, selection bias, and other sources of systematic error inherent in non‐randomised research. Second, the number of available studies was relatively limited, reducing overall statistical power, the robustness of the pooled estimates, and the potential for subgroup analyses. Third, differences in study populations, including disease activity, treatment status, duration of acromegaly, body composition, and metabolic comorbidities, may have contributed to between‐study heterogeneity despite the use of random‐effects models. Furthermore, although MRS and MRI‐PDFF are considered the most accurate noninvasive techniques for quantifying hepatic fat content, methodological differences in MRI acquisition protocols and fat quantification thresholds across studies may have introduced variability [37]. Liver stiffness was used as a noninvasive surrogate for hepatic fibrosis rather than as a substitute for histological confirmation. Although VCTE, MRE, and other elastography methods are widely accepted for fibrosis assessment, liver stiffness measurements may be influenced by factors unrelated to hepatic fibrosis, such as hepatic inflammation, vascular congestion, or changes in liver tissue composition, potentially leading to over‐ or underestimation of the true burden of hepatic fibrosis [37]. Finally, although we used a random‐effects model, caution is warranted when interpreting certain results given the relatively high heterogeneity observed in specific analyses.

Notwithstanding these limitations, our study has several strengths. Specifically, we applied stringent inclusion criteria, limiting eligibility to studies that quantified hepatic fat content with MRI‐based techniques and assessed liver stiffness with well‐validated noninvasive methods. By focussing on these validated imaging modalities, we believe our findings provide reliable and robust estimates of associations among acromegaly, disease activity, and hepatic fat accumulation.

In conclusion, this meta‐analysis shows that adult patients with acromegaly have significantly lower MRI‐assessed hepatic fat content than matched controls, particularly during active disease, whereas no significant differences in liver stiffness were observed. These findings suggest a potential protective or modulatory effect of GH excess on hepatic steatosis (MASLD) and indicate no significant burden of hepatic fibrosis, as assessed by non‐invasive elastography methods. Future research should prioritise well‐designed longitudinal studies to assess the evolution of MASLD/MASH and liver‐related clinical events before and after achieving biochemical control of acromegaly. These studies should incorporate standardised quantitative liver imaging, detailed assessments of insulin sensitivity and body fat distribution, and comprehensive characterisation of treatment modalities and disease duration. In addition, mechanistic studies are needed to clarify the molecular pathways by which excess GH promotes insulin resistance while protecting against hepatic fat accumulation. Finally, given the potential association between alterations in the GH/IGF‐1 axis and hepatocarcinogenesis [48], further studies should determine whether changes in hepatic fat content and fibrosis after acromegaly treatment translate into differences in long‐term risk of advanced liver disease and hepatocellular carcinoma.

## Author Contributions

A.M. and G.T. contributed to study conception, analysis, and interpretation of the results. A.M. and G.T. wrote the first draft of the manuscript. A.M., R.M., E.M., D.M., N.R., M.F., and M.Z. contributed to study conduct and literature searches. S.P. contributed to interpreting the results and to the discussion. All authors edited, reviewed, and approved the final version of the manuscript. A.M. is the guarantor of this work and, as such, has full access to all study data and is responsible for its integrity and the accuracy of the analysis.

## Funding

The authors have nothing to report.

## Conflicts of Interest

The authors declare no conflicts of interest.

## Supporting information


Supporting Information S1

## Data Availability

The data that supports the findings of this study are available in the supplementary material of this article.
