# Supplementary material for: Metabolic Dysfunction‐Associated Steatotic Liver Disease in Adults With Acromegaly: A Meta‐Analysis of Observational Studies
Source: Diabetes Metab Res Rev. 2026 Sep 21;42(6):e70231. doi: 10.1002/dmrr.70231 (PMC13591288; doi:10.1002/dmrr.70231)
Supplement: Supplementary file 1 [file DMRR-42-e70231-s001.docx]

**ONLINE-ONLY SUPPLEMENTARY MATERIAL**

**Supplementary Table 1**. Syntax used for database searches on PubMed and Scopus.

PubMed <up to June 15, 2026> (*restriction terms*: Human and English).

| #1 | Acromegaly AND fatty liver | 16 |
| --- | --- | --- |
| #2 | Acromegaly AND non-alcoholic fatty liver disease | 4 |
| #3 | Acromegaly AND non-alcoholic steatohepatitis | 4 |
| #4 | Acromegaly AND NAFLD | 4 |
| #5 | Acromegaly AND NASH | 3 |
| #6 | Acromegaly AND metabolic dysfunction associated steatotic liver disease | 5 |
| #7 | Acromegaly AND metabolic associated steatohepatitis | 11 |
| #8 | Acromegaly AND MASLD | 1 |
| #9 | Acromegaly AND MASH | 0 |

Scopus <up to June 15, 2026> (*restriction terms*: Human and English).

| #1 | Acromegaly AND fatty liver | 40 |
| --- | --- | --- |
| #2 | Acromegaly AND non-alcoholic fatty liver disease | 11 |
| #3 | Acromegaly AND non-alcoholic steatohepatitis | 1 |
| #4 | Acromegaly AND NAFLD | 5 |
| #5 | Acromegaly AND NASH | 0 |
| #6 | Acromegaly AND metabolic dysfunction associated steatotic liver disease | 3 |
| #7 | Acromegaly AND metabolic associated steatohepatitis | 0 |
| #8 | Acromegaly AND MASLD | 2 |
| #9 | Acromegaly AND MASH | 0 |

**Supplementary Table 2**. Observational studies excluded at the eligibility stage (see the PRISMA flow diagram).

| Author, Year (Ref) | Main Reason(s) |
| --- | --- |
| Szendroedi *et al.* 2008 (26) | Unsatisfactory study design |
| Koutsou-Tassopoulou *et al.* 2019 (27) | Only patients with acromegaly (absence of a control group) |

Note: The reference number reported in parentheses corresponds to the reference list of the manuscript.

**Supplementary Table 3**. Newcastle–Ottawa Scale (NOS) for studies included in the meta-analysis.

| Author, Year (Ref.) | Selection | Comparability | Outcome | Total |
| --- | --- | --- | --- | --- |
| Winhofer et al. 2014 (28) | *** | ** | ** | 7 |
| Bredella et al 2017 (29) | *** | ** | ** | 7 |
| Fellinger et al. 2020 (30) | ** | ** | ** | 6 |
| Kuker et al. 2021 (31) | ** | ** | ** | 6 |
| Eroglu et al. 2023 (32) | *** | ** | ** | 7 |
| Arlien-Søborg et al. 2023 (33) | *** | ** | ** | 7 |
| Bankir et al. 2019 (34) | *** | ** | * | 6 |
| Coskum et al. 2024 (35) | *** | ** | * | 6 |
| Apaydin et al. 2026 (36) | *** | ** | * | 6 |

Note: The reference number reported in parentheses corresponds to the reference list of the manuscript.

**Supplementary Figure 1**. The PRISMA flow diagram summarizes the search and selection processes for the meta-analysis.

**Supplementary Figure 2**. Leave-one-out sensitivity analysis to assess the influence of each eligible study on the overall effect-size estimate.

**Supplementary Figure 3**. Univariable meta-regression assessing the association between age and the weighted mean difference (WMD) in hepatic fat content between patients with acromegaly and control subjects (p=0.612).

**Supplementary Figure 4**. Univariable meta-regression assessing the association between body mass index and the weighted mean difference (WMD) in hepatic fat content between patients with acromegaly and control subjects (p=0.264).

**Supplementary Figure 5**. Univariable meta-regression assessing the association between sex and the weighted mean difference (WMD) in hepatic fat content between patients with acromegaly and control subjects (p=0.994).

**Supplementary Figure 6**. Univariable meta-regression assessing the association between HOMA-IR score and the weighted mean difference (WMD) in hepatic fat content between patients with acromegaly and control subjects (p=0.822).

**Supplementary Figure 7.** Univariable meta-regression assessing the association between circulating IGF-1 levels and the weighted mean difference (WMD) in hepatic fat content between patients with active acromegaly and those with controlled acromegaly (p=0.002).

**Supplementary Figure 8**. The DOI plot for studies comparing hepatic fat content among patients with active or controlled acromegaly and control subjects.
